# Handwriting speed and pen motor control in older adults with and without cognitive impairment

**DOI:** 10.3389/fnhum.2026.1820193

**Published:** 2026-05-20

**Authors:** João Galrinho, Orlando Fernandes, Ana Rita Silva, Marta A. Gonçalves-Montera, Ana Rita Matias

**Affiliations:** 1 Department of Sports and Health, School of Health and Human Development, Universidade de Évora, Évora, Portugal; 2 Department of Sports and Health, School of Health and Human Development, Comprehensive Health Research Centre Polo di Evora, Evora, Portugal; 3 Faculty of Psychology, Universidade de Lisboa, Lisbon, Portugal

**Keywords:** cognitive aging, digital biomarkers, elderly, institutionalized older adults, writing, digital handwriting analysis, process-product relationship

## Abstract

**Background:**

Handwriting is a hierarchical cognitive–motor activity requiring the integration of motor execution, visuospatial processing, working memory, and executive control. Digital handwriting technology enables simultaneous assessment of process (kinematics) and product (performance outcomes), offering a theoretically grounded approach to detecting cognitive vulnerability in aging.

**Methods:**

This study examined whether kinematic handwriting features differentiate institutionalized older adults with and without cognitive impairment and whether these features predict handwriting product performance under varying cognitive–motor demands. Fifty-eight participants (20 cognitively healthy; 38 cognitively impaired), classified using education-adjusted MMSE cutoffs, completed pen-control tasks (DOTS, LINES) and four handwriting-speed tasks (two copy, two dictation) on a digitizing tablet. Nine standardized kinematic variables were analyzed using logistic and multiple linear regression models with correction for multiple comparisons.

**Results:**

Pen-control tasks (DOTS, LINES) did not significantly discriminate between the two groups, the handwriting-speed tasks, particularly dictation, revealed significant group differences. Temporal efficiency and stroke organization variables (e.g., Duration, Number of Strokes) significantly contributed to classification in high-demand tasks. Among cognitively healthy participants, associations between kinematic and product measures were limited, suggesting preserved compensatory mechanisms. Conversely, cognitively impaired individuals exhibited stronger process–product coupling, with Start Time, Vertical Size, and Duration significantly predicting handwriting performance in dictation tasks.

**Conclusion:**

Handwriting kinematics, especially temporal and stroke-related features, are sensitive indicators of cognitive impairment when assessed under high cognitive–motor load. These findings support the use of digitally mediated handwriting tasks—particularly dictation paradigms—as ecologically valid, low-cost tools for screening and monitoring cognitive decline in older adults.

**Clinical trial registration:**

ClinicalTrials.gov, NCT06483438.

## Highlights

- Diagnostic differentiation emerged only in tasks with higher cognitive–motor demands.
- Temporal efficiency and stroke organization were the most robust predictors of cognitive impairment.
- Process–product coupling was minimal in healthy aging but pronounced in cognitive impairment.
- Dictation tasks showed greater sensitivity than isolated pen-control tasks.
- Digital handwriting analysis offers a scalable, non-invasive approach for early cognitive screening and longitudinal monitoring.

## 1 Introduction

Handwriting is a complex neurocognitive activity that integrates fine motor control, perceptual processing, language systems, and executive functions. Due to its multidimensional nature, it has increasingly been recognized as a potentially sensitive marker of cognitive functioning in older adults. Age-related changes in neuromuscular and central nervous system function often lead to slower and less fluent handwriting, reflecting declines in motor control, visuomotor integration, and processing speed (Salthouse, 2019; Seidler et al., 2010). These changes are typically more pronounced in individuals with cognitive impairment, including those with mild cognitive impairment (MCI) and early-stage neurodegenerative conditions such as Alzheimer’s disease (AD) and Parkinson’s disease (PD). As a result, distinguishing between handwriting features associated with normal aging and those linked to pathological decline has become an important focus in clinical and gerontological research.

Early studies showed that handwriting characteristics in older adults are shaped not only by musculoskeletal aging but also by cognitive deterioration, allowing differentiation between individuals with and without cognitive impairment (Liu, 2011). Subsequent research expanded this work by examining features such as tremor patterns, stroke regularity, pressure variability, and narrative handwriting in various neurodegenerative and movement disorders (Schiegg and Thorpe, 2017). These studies consistently demonstrate that cognitive impairments—particularly in executive functioning, working memory, and visuospatial processing—are reflected in handwriting through reduced fluency, irregular stroke patterns, and impaired motor planning (Rosenblum et al., 2010; Rosenblum et al., 2013).

Advances in digital technology have enabled precise, real-time analysis of handwriting using kinematic features such as stroke duration, velocity, jerk, pen pressure, and spatial amplitude. Tablet-based systems have proven effective at detecting subtle motor abnormalities associated with cognitive decline (Impedovo et al., 2014). For instance, Diaz et al. (2021) and Faundez-Zanuy et al. (2021) demonstrated that machine learning models can accurately classify handwriting produced by individuals with PD or AD. More recently, hybrid machine-learning approaches have demonstrated promising diagnostic accuracy for AD by combining spatiotemporal handwriting features with cognitive screening test results, highlighting the potential of multimodal digital biomarkers (Demircioglu Diren, 2025). These developments underline the clinical promise of handwriting as a non-invasive, accessible, and ecologically valid tool for early detection of cognitive impairment.

A growing number of systematic reviews support the diagnostic value of handwriting and drawing tasks. Yamada et al. (2022) reported that both paper-based and digital drawing tests reliably distinguish healthy aging from MCI and early AD, especially when tasks require visuoconstruction, planning, and sustained motor control. Similarly, studies across the adult lifespan have identified both stable and age-dependent handwriting traits, emphasizing the need to differentiate normal motor aging from pathological changes (Faundez-Zanuy et al., 2021). Taking together, current evidence suggests that handwriting reflects the interaction between cognitive and motor systems and may serve as an early behavioral marker of cognitive vulnerability.

Recent research increasingly supports assessing both the handwriting process (i.e., kinematics) and product (e.g., speed, legibility, accuracy) in older adults. Studies using digitizing pens and tablets have shown that temporal and spatial kinematic features—such as velocity, pressure, pause frequency, and stroke organization—are systematically associated with handwriting performance, frailty status, and cognitive function in both community-dwelling and “young-old” populations (Camicioli et al., 2015; Engel-Yeger et al., 2012; van Drempt et al., 2011). Kinematic analyses of handwriting and drawing tasks have also distinguished clinical populations (e.g., mild depression, prodromal and clinical dementia) even when traditional product scores remain unaffected, highlighting the added value of process-based measures (Heinik et al., 2010; Yamada et al., 2022). More recently, machine learning studies have used real-time handwriting data to classify age groups and detect MCI, leveraging both process and product variables (Asci et al., 2022; Bednorz et al., 2025; Toffoli et al., 2025). These findings reinforce the utility of a combined process–product approach in aging research: while product outcomes reflect functional performance, kinematic features reveal subtle motor–cognitive changes that may otherwise go undetected.

Despite the growing body of evidence, several limitations persist. Many studies rely on single handwriting tasks, limiting the ability to assess how different task demands affect motor–cognitive performance. There is also wide variability in protocols and kinematic variables, making comparisons across studies difficult. Moreover, research on institutionalized older adults remains limited, despite this group’s high rates of cognitive impairment and diverse functional profiles. Additionally, the relationship between handwriting process variables (e.g., timing, spatial control) and product outcomes (e.g., legibility, speed) is still poorly understood. Determining whether kinematic features can predict functional handwriting performance is essential for establishing their clinical relevance.

Contemporary models conceptualize handwriting not as a purely motor act but as a multicomponent process in which transcription is coordinated within working memory systems that simultaneously manage graphomotor execution, orthographic retrieval, phonological processing, and syntactic (Berninger, 2009; De Vita et al., 2021; Olive and Kellogg, 2002). This framework suggests that handwriting tasks differ systematically in cognitive demand depending on the extent to which they engage linguistic transformation and executive control mechanisms.

Motor control paradigms, such as dot-and-line tasks, are designed to isolate graphomotor execution from higher linguistic demands. These tasks primarily require visuomotor coordination, spatial accuracy, temporal regulation, and continuous online monitoring of movement parameters, while minimizing phonological and orthographic processing. Because stimuli are simple and externally specified, cognitive load is largely restricted to sensorimotor integration and motor planning. In aging research, this distinction is critical: declines in fine motor control and processing speed may occur independently of cognitive impairment, and motor-only tasks provide a baseline against which cognitively demanding handwriting tasks can be contrasted (Impedovo and Pirlo, 2019; Stefano et al., 2019). If group differences are limited in dots and lines but amplified in linguistically mediated tasks, this pattern would suggest that executive and working memory mechanisms—rather than pure motor dysfunction—drive performance divergence.

Copying and dictation tasks introduce progressively greater cognitive demands. Within working memory theory, transcription requires coordination among the phonological loop, the visuospatial system, and the central executive (De Vita et al., 2021; Olive and Kellogg, 2002). When handwriting movements are automatized, motor execution consumes fewer cognitive resources, allowing greater allocation of cognitive resources to higher-order linguistic processes. However, reduced automatization—as may occur in cognitive aging—results in increased competition for limited working memory capacity (De Vita et al., 2021; Truxius et al., 2024).

Copying from a visual model primarily involves visual analysis of letter forms, short-term visuospatial retention, and motor reproduction. Because orthographic representations are externally available, demands on phonological decoding and orthographic retrieval are comparatively low. Executive control primarily coordinates attention and motor execution. Dictation, by contrast, requires online phonological decoding of auditory input, phoneme–grapheme conversion, orthographic selection, and serial maintenance of linguistic material during motor production. Sentence-level dictation further increases demands by requiring syntactic structuring and the maintenance of partially processed lexical units, thereby intensifying reliance on updating, inhibition, and cognitive shifting processes (Suárez-Coalla et al., 2025; Vessio, 2019). Empirical research consistently demonstrates that tasks involving greater linguistic transformation and orthographic uncertainty increase working memory load and reduce motor fluency and spatial precision (De Vita et al., 2021; Fernandes et al., 2023; Olive and Kellogg, 2002).

This graded cognitive architecture is particularly relevant in the context of aging and neurodegenerative conditions. Dynamic handwriting analyses in Alzheimer’s and Parkinson’s disease indicate that tasks requiring concurrent motor and linguistic processing amplify performance differences between clinical and control groups (Impedovo and Pirlo, 2019; Stefano et al., 2019; Vessio, 2019). Sentence writing under dictation has been characterized as highly sensitive to limitations in working memory and executive control. Machine-learning analyses of stroke-level kinematic features further support the discriminative value of dictation paradigms for detecting subtle cognitive decline (Nardone et al., 2025).

Overall, motor control tasks (such as dots and lines) primarily assess foundational graphomotor execution, copying introduces moderate visuospatial and attentional demands, and dictation imposes substantial executive and working memory load through phonological–orthographic transformation and serial maintenance. Conceptualizing handwriting within this hierarchical framework allows kinematic performance to be interpreted as a behavioral marker of the interaction between motor systems and higher-order cognitive control mechanisms, providing a theoretically grounded basis for examining differentiation between normal cognitive aging and cognitive impairment.

### 1.1 The present study

The present study addresses critical gaps in the literature by investigating whether handwriting speed and pen motor control can distinguish older adults with cognitive impairment from their cognitively healthy, institutionalized peers. By identifying which process-based (kinematic) and product-based (performance outcome) indicators are most sensitive to cognitive decline, this research contributes to the development of a more comprehensive and accurate framework for handwriting assessments that supports earlier detection of functional changes and enables more targeted therapeutic interventions.

Participants completed standardized pen-control tasks (DOTS and LINES) and multiple handwriting-speed tasks (WS1–WS4). Both kinematic process variables and handwriting product indicators were analyzed. By comparing performance across tasks with varying cognitive-motor demands, the study explores whether specific features of handwriting execution-such as stroke duration, movement amplitude, pressure variability, and movement smoothness- can serve as reliable markers of cognitive impairment in older adults.

Accordingly, this study addresses the following questions: (1) Can handwriting speed and pen motor control effectively differentiate older adults with cognitive impairment from those with normal cognitive aging? (2) Which kinematic features are independently associated with motor control (DOTS and LINES) and handwriting products (e.g., handwriting speed in copy and dictation)?

## 2 Methods

### 2.1 Sampling and procedures

This study included 58 institutionalized older adults, both with (*n* = 38) and without (*n* = 20) cognitive impairment on the Mini-Mental State Examination (MMSE). The mean age in the cognitively impaired group was 86.05 years (SD = 6.26), with ages ranging from 62 to 99 years. In the group without cognitive impairment, the mean age was 84.35 years (SD = 7.78), ranging from 67 to 99 years.

In terms of gender distribution, the group with cognitive impairment included 14 men (36.8%) and 24 women (63.2%). The group without impairment consisted of 5 men (25%) and 15 women (75%).

Participants with cognitive impairment were included in the study if they were aged 60 years or older, had cognitive impairment confirmed by medical records, and scored below established thresholds on the MMSE, adjusted for education level based on Portuguese normative data (Morgado et al., 2010). Specifically, the cutoff scores were ≤15 for illiterate individuals, ≤22 for those with 1–11 years of education, and ≤27 for those with more than 11 years of education. Additionally, participants in this group had to score 6 or lower on the Clock Drawing Test (Morgado et al., 2010).

For participants without cognitive impairment, inclusion required clinical documentation indicating that institutionalization was not due to cognitive decline, but rather for other reasons such as rehabilitation, social isolation, or accompanying an institutionalized spouse. These individuals also needed to score above the MMSE cut-off thresholds for their education level (i.e., >15 for illiterate individuals, >22 for those with 1 to 11 years of education, and >27 for those with more than 11 years of education) and obtain a score higher than 6 on the Clock Drawing Test (Morgado et al., 2010).

Exclusion criteria were applied to both groups to reduce potential confounding factors that might affect handwriting or cognitive performance independently of cognitive status. Participants were excluded if they had a history of neurological or psychiatric disorders unrelated to normal aging (such as stroke, Parkinson’s disease, Huntington’s disease, major depression, or psychosis) that could influence motor or cognitive outcomes. Other exclusion criteria included severe sensory or motor impairments (e.g., uncorrected visual deficits or upper-limb paralysis) that could interfere with the testing protocol or data collection, as well as non-native Portuguese speakers, which could compromise the validity of the verbal cognitive assessment tools used.

Although medication use was not controlled experimentally, medical records were reviewed to identify major neurological or psychiatric conditions and pharmacological treatments known to produce significant motor side effects (e.g., dopaminergic medication, antipsychotics). No participants presented clinical conditions or treatments expected to severely compromise handwriting motor performance.

### 2.2 Ethical approval

This study received approval from an institutional Ethics Committee and Scientific Council (Reference No. GD/28695/2023) prior to data collection. Participants were pre-screened using institutional medical records to confirm cognitive status prior to inclusion. If unexpected cognitive difficulties had been identified during screening, the institutional clinical staff responsible for the participant’s care would have been informed to allow appropriate clinical follow-up. The informed consent includes permission to use anonymized participant data in scientific publications and the right for participants to withdraw without any penalty. For participants whose cognitive abilities were inconsistent, their verbal consent was sought several times during the assessment. After approval, the entities were contacted to present the project and begin contacting the participants. Then, each participant (or their legal tutor) provided informed consent, stating that they were aware of the confidentiality of the data collected throughout the study and that it would not be disclosed and would be used only for academic purposes.

### 2.3 Instruments

All assessment procedures were registered as a protocol on ClinicalTrials.gov (identifier: NCT06483438). Cognitive impairment screening was conducted using standardized instruments. The neuropsychological assessment was administered by a Psychology undergraduate student and the principal investigator, under the supervision of a professional with advanced training in Neuropsychology. To identify cognitive impairment, three sources of information were used: (i) the Mini-Mental State Examination (MMSE), a widely used cognitive screening tool that assesses global cognitive function across six domains with 30 items (Morgado et al., 2010); (ii) the Clock Drawing Test, commonly used in neurological, psychiatric, and psychological assessments, and increasingly adopted for detecting age-related cognitive changes (Pinto, 2022). The scoring method applied followed the criteria established by Shulman (2000); and (iii) medical records confirming cognitive impairment, including clinical documentation, neuropsychological assessments, and behavioral observations in daily routines.

**Figure 1:**
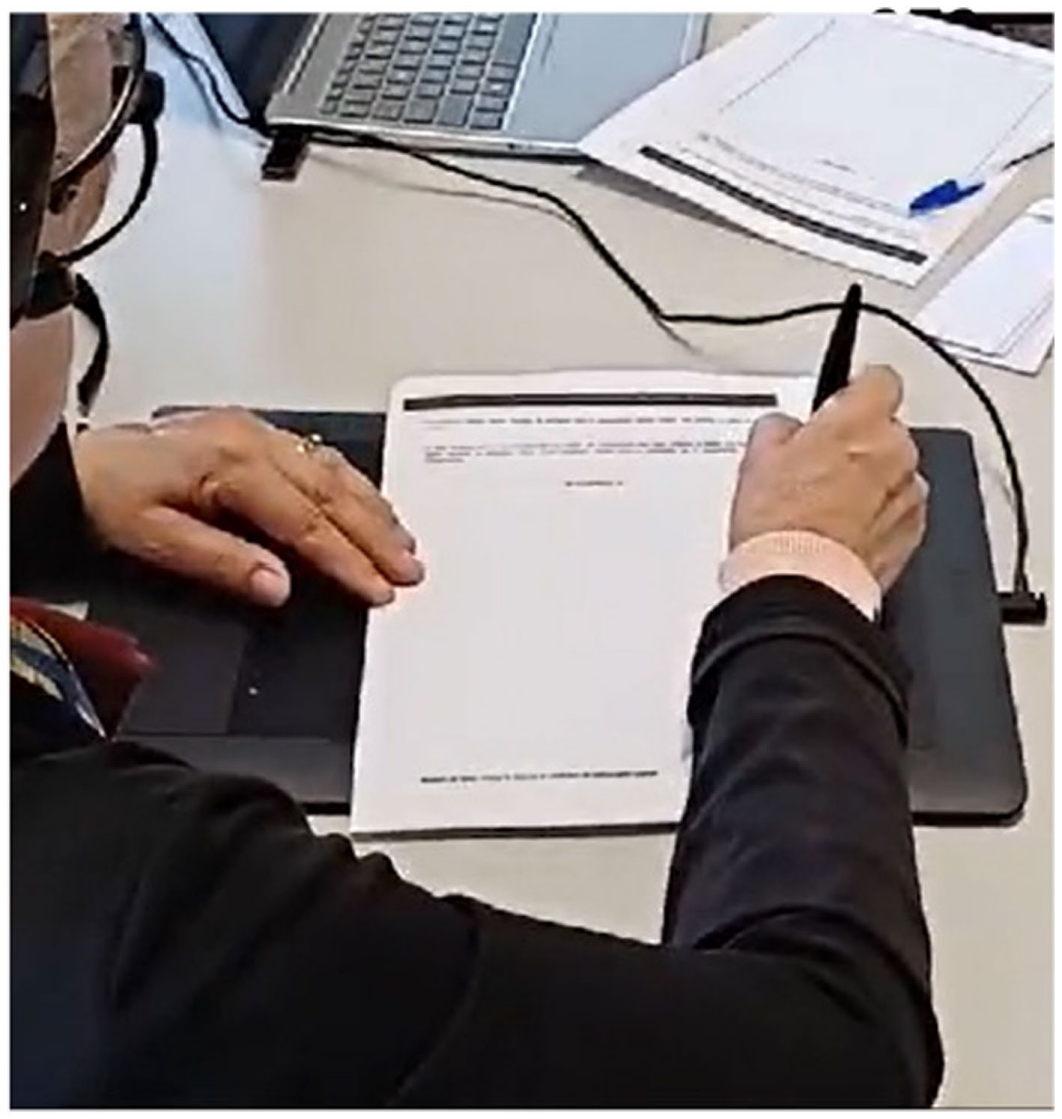
Assessment setting.

Handwriting assessments were carried out by a psychomotor therapist with extensive experience in the field. Participants were invited to sit comfortably in a chair and experiment with the pen on the table and its position on the digitizing tablet (Figure 1).

Two subtests from the Handwriting Assessment Battery (HAB) were used in this study to assess pen control (e.g., drawing lines and dots within a specified time) and handwriting speed (e.g., copying a sentence under time constraints).

Pen control was assessed using two tasks. In the first task (referred to as LINES), participants were instructed to draw at least 10 horizontal lines within 20 s. In the second task (DOTS), they were required to make at least 10 dots on a sheet of paper within the same time frame. For both tasks, participants were given three attempts, and performance on each attempt was recorded dichotomously (achieved/not achieved). The onset of movement was defined as the first pen-down event detected by the digitizing tablet following stimulus presentation. Pen-down and pen-up events were automatically recorded and used by the MovAlyzeR software to segment strokes and compute kinematic variables.

Handwriting speed was evaluated using four tasks: two copying and two dictation exercises (McCluskey and Lannin, 2003). The sequence of tasks was as follows: first, copying the sentence *O João viu o camião vermelho* (WS1) from a card shown; second, dictating the same sentence (WS2); third, dictating the sentence. *Os peixes retiravam o ar da água* (WS3); and fourth, copying the same sentence (WS4) from a card shown. For each task (WS1–WS4), the outcome was recorded as a dichotomous indicator of whether the participant met the HAB criterion for average characters per minute.

Both motor control (DOTS and LINES) and handwriting speed tasks (WS1 to WS4), were performed on a on an A4 sheet placed on thean x-y digitizing tablet (WACOM, Intuos Pro L, model PTH-851-PT) with an inking pen (WACOM Grip Pen, model KP-501E). Also, NeuroScript’s MovAlyzeR software (version 6.1) run on a MS Windows laptop computer connected to the tablet via USB (NeuroScript, 2023). The tablet had an active surface area of 32.51 cm x 20.32 cm, a device resolution of 0.0005 cm, and a sampling frequency of 100 Hz. Kinematic variables were automatically computed by the MovAlyzeR software using established algorithms for digital handwriting analysis. Although the detailed computational procedures are beyond the scope of the present manuscript, key measures such as normalized jerk have been extensively described and validated in previous research (Teulings et al., 1997; Van Gemmert et al., 2001).

The following features were extracted using MovAlyzeR (NeuroScript, 2020):

- Vertical and horizontal size: the distance in centimeters between the start and end points of a stroke, measured vertically and horizontally.
- Slant: the direction of a stroke in radians, from its start to end point. A horizontal rightward stroke equals 0 radians; 60° to the right equals 1.05 radians; and 90° (upright) equals 1.57 radians.
- Start time: the reaction time, defined as the time (in seconds) between stimulus onset and the start of the first stroke.
- Duration: the time interval (in seconds) between the first and last data points of a stroke or segment.
- Relative pen-down duration: the proportion of time the pen remained in contact with the writing surface during the stroke.
- Normalized jerk: a unitless measure of movement smoothness, calculated by normalizing jerk with respect to stroke duration and size.
- Average pen pressure: the mean axial force applied to the pen, expressed in pen tablet units. A value of 400 corresponds to approximately 100 grams of force.
- Number of strokes: the count of individual upward or downward pen strokes produced during handwriting.

Kinematic variables were computed based on pen-down segments (individual strokes) automatically identified by the digitizing system. For each task, stroke-level measures were calculated and aggregated (mean values) to obtain participant-level kinematic indicators.

### 2.4 Data analysis

All statistical analyses were conducted using Jamovi (Version 2.4), employing the ANOVA, RStats, and Exploration modules. Prior to hypothesis testing, assumptions of normality and sphericity were evaluated using the Shapiro–Wilk and Mauchly’s tests, respectively. Several kinematic variables violated normality; therefore, descriptive statistics were reported as medians and interquartile ranges for non-normally distributed variables and as means and standard deviations when appropriate. Non-parametric effect sizes were calculated using *r* = Z/√N and interpreted according to Cohen’s benchmarks (*r* ≈ 0.10 small, 0.30 medium, 0.50 large). Statistical significance was initially set at α = 0.05. However, Bonferroni correction was applied in analyses involving multiple simultaneous comparisons within the same model (e.g., multiple predictors entered concurrently in regression models) to control for Type I error inflation. In these cases, adjusted significance thresholds were defined as *p* < 0.05 divided by the number of predictors tested within that specific model. Because aging-related motor slowing may influence handwriting kinematics independently of cognitive status, analyses focused on relative differences across tasks with varying cognitive–motor demands rather than absolute motor speed alone. This approach reduces the likelihood that observed effects are attributable solely to general motor slowing or medication-related motor effects.

In the regression analyses, kinematic measures were treated as process variables (predictors), whereas product variables corresponded to task performance outcomes. For the motor-control tasks (DOTS and LINES), the product variable indicated whether the participant met the predefined HAB performance criterion (achieved vs. not achieved). For the handwriting-speed tasks (WS1–WS4), the product variable corresponded to handwriting speed, operationalized as characters produced per minute.

To examine whether kinematic features differentiated older adults with and without cognitive impairment, a series of binary logistic regression models was performed. Diagnostic group (0 = without cognitive impairment; 1 = with cognitive impairment) was entered as the dependent variable, and nine standardized kinematic predictors (process variables) were included: Vertical Size, Horizontal Size, Slant, Start Time, Duration, Relative Pen-Down Duration, Normalized y-Jerk, Average Pen Pressure, and Number of Strokes. Missing data (<5%) was imputed using the median prior to analysis. Model fit was evaluated using Deviance, Akaike Information Criterion (AIC), and Cragg–Uhler/Nagelkerke pseudo-R^2^ indices. The statistical significance of individual predictors was assessed using Wald z tests, applying Bonferroni-adjusted alpha levels within each regression model to account for the number of predictors entered simultaneously. Odds ratios were interpreted as indicators of each predictor’s unique contribution to diagnostic classification. Additional binary logistic regressions were conducted separately for the DOTS and LINES tasks (motor control tasks) to determine whether the same kinematic predictors were associated with product-based task outcomes, with correction applied analogously within each task-specific model.

Multiple linear regression analyses were subsequently performed to investigate the relationship between kinematic process variables and handwriting product performance in the handwriting-speed tasks. Regression modeling was selected to estimate the independent contribution of multiple continuous predictors while providing interpretable effect sizes. Although some predictors deviated from normality, regression techniques are considered robust to moderate violations when predictors are standardized and the sample size exceeds 30 (Schmidt and Finan, 2018). Prior to estimation, multicollinearity diagnostics were examined, with all Variance Inflation Factor (VIF) values remaining below 5. For linear models, residual assumptions were evaluated through visual inspection of Q–Q plots and residual-versus-fitted scatterplots, which indicated approximate normality and no systematic heteroscedasticity. A Bonferroni correction was applied to the set of regression coefficients within each linear model when interpreting the significance of individual predictors. A post-hoc power analysis was conducted to assess sample adequacy. Considering the total sample (*N* = 58), α = 0.05, and a medium effect size (*f*^2^ ≈ 0.15 / OR ≈ 1.8), estimated statistical power for the primary regression models exceeded 0.80. Although subgroup analyses involved smaller samples, power remained acceptable for detecting medium-to-large effects, indicating sufficient sensitivity to identify clinically meaningful associations while acknowledging reduced sensitivity for small effects. Given the smaller size of the cognitively healthy subgroup (*n* = 20), the regression analyses are primarily powered to detect moderate to large effects and should be interpreted with appropriate caution.

Diagnostic group was entered as the dependent variable in logistic regression models to evaluate the discriminative capacity of handwriting kinematic features, a common approach in biomarker research aimed at identifying indicators associated with clinical classification.

## 3 Results

### 3.1 Handwriting speed and pen-control kinematics as indicators distinguishing older adults with and without cognitive impairment

A series of binomial logistic regression analyses was conducted to evaluate whether kinematic features from handwriting speed tasks (WS1–WS4) and pen-control tasks (DOTS and LINES) could differentiate older adults with cognitive impairment from those with typical cognitive aging. The DOTS model was not statistically significant, χ^2^(9) = 6.26, *p* = 0.714, explaining a limited proportion of variance (*R*^2^*_CS_* = 0.122; Nagelkerke *R*^2^ = 0.163). Similarly, for the LINES task, none of the kinematic predictors reached statistical significance, and the model explained only a modest proportion of variance (*R*^2^*_CS_* = 0.283; Nagelkerke *R*^2^ = 0.377). Also, models for WS1 (copy) (χ^2^(9) = 8.16, *p* = 0.518; *R*^2^*_CS_* = 0.159) and WS2 (dictation) (χ^2^(9) = 5.94, *p* = 0.746; *R*^2^*_CS_* = 0.119) were not statistically significant and showed low explanatory capacity. The WS3 (dictation) model yielded the strongest results, χ^2^(9) = 20.20, *p* = 0.017, explaining a substantial proportion of variance (*R*^2^*_CS_* = 0.349; Nagelkerke *R*^2^ = 0.465). Within this model, Duration (*b* = 18.08, *p* = 0.021) and Number of Strokes (*b* = 0.13, *p* = 0.006) significantly increased the likelihood of cognitive impairment, while Vertical Size (*p* = 0.056) and Start Time (*p* = 0.051) showed marginal effects. The WS4 (copy) model was not statistically significant, χ^2^(9) = 7.93, *p* = 0.541, despite moderate variance explained (*R*^2^*_CS_* = 0.172; Nagelkerke *R*^2^ = 0.231), although Duration (*b* = 14.11, *p* = 0.029) and Number of Strokes (*b* = 0.08, *p* = 0.032) emerged as significant predictors. Overall, these findings indicate that only specific kinematic features related to temporal execution and stroke segmentation, particularly in WS3, meaningfully contributed to differentiating cognitive impairment status, whereas pen-control variables and other handwriting tasks showed limited discriminatory power (see Table 1).

**Table 1:** Binary logistic regression models predicting cognitive impairment from kinematic features across pen-control and handwriting-speed tasks.

Task	χ^2^	df	*p*	Cox & Snell *R*^2^	Nagelkerke *R*^2^
DOTS	6.26	9	0.714	0.122	0.163
LINES	17.60	9	0.040	0.283	0.377
WS1 copy	8.16	9	0.518	0.159	0.212
WS2 dictation	5.94	9	0.746	0.119	0.158
WS3 dictation	**20.20**	**9**	**0.017**	**0.349**	**0.465**
WS4 copy	7.93	9	0.541	0.172	0.231

χ^2^ values represent the omnibus likelihood-ratio tests for each model. df = degrees of freedom. Cox & Snell and Nagelkerke *R*^2^ values represent pseudo-*R*^2^ effect size estimates. Bold values indicates statistically significant results (*p* < 0.05).

### 3.2 Association between handwriting process (kinematic features) and the handwriting product (handwriting speed) of individuals during pen-control and handwriting tasks (copy and dictation)

#### 3.2.1 Motor control tasks (DOTS and LINES)

Separate binary logistic regression analyses were conducted for the DOTS and LINES tasks to assess whether nine standardized process variables—Vertical Size, Horizontal Size, Slant, Start Time, Duration, Relative Pen-Down Duration, Normalized y-Jerk, Average Pen Pressure, and Number of Strokes—predicted performance on two corresponding product variables: DOTS (achieved or not) and LINES (achieved or not). Prior to analysis, missing values were imputed using the median, and all predictors were standardized.

As shown in Tables 2, 3, none of the models were statistically significant. These results suggest that the kinematic process variables did not meaningfully predict handwriting outcomes in the DOTS and LINES tasks. In other words, the temporal and spatial features of pen movement were not strongly associated with task performance as measured by product-based criteria.

**Table 2:** Binary logistic regression predicting handwriting product variables from DOTS kinematic predictors.

Outcome variable	χ^2^(df = 9)	*p*	Nagelkerke *R*^2^
LINES	0.61	1.000	0.07
DOTS	9.92	0.357	0.22

No individual predictor reached significance at α = 0.05.

**Table 3:** Binary logistic regression predicting handwriting product variables from LINES kinematic predictors.

Outcome variable	χ^2^(df = 9)	*p*	Nagelkerke *R*^2^
LINES	1.57	0.997	0.17
DOTS	6.11	0.729	0.14

No individual predictor reached significance at α = 0.05.

#### 3.2.2 Handwriting tasks

##### 3.2.2.1 Group without cognitive impairment

To examine whether kinematic process variables could predict handwriting product performance (i.e., handwriting speed) in older adults without cognitive impairment, four multiple linear regression analyses were conducted, one for each handwriting task (WS1–WS4). In each model, nine kinematic predictors were entered simultaneously: Vertical Size, Horizontal Size, Slant, Start Time, Duration, Relative Pen-Down Duration, Normalized y-Jerk, Average Pen Pressure, and Number of Strokes. For WS1 (copy), the overall regression model was not statistically significant (*R* = 0.63, *R*^2^ = 0.40), and none of the individual predictors reached significance (all *p*s > 0.10). The Number of Strokes showed a non-significant trend toward slower handwriting (*p* = 0.106). The WS2 (dictation) model also failed to reach statistical significance, despite a high proportion of explained variance (*R* = 0.87, *R*^2^ = 0.76). No individual kinematic variable significantly contributed to this model (all *p*s > 0.10). In contrast, the WS3 (dictation) model was statistically significant (*R* = 0.90, *R*^2^ = 0.81). In this model, Horizontal Size emerged as a strong predictor of slower handwriting speed (*b* = 711.79, *t* = 3.65, *p* = 0.003), while Normalized y-Jerk approached significance (*p* = 0.073), suggesting a potential association with handwriting fluency. For WS4 (copy), the model was not statistically significant (*R* = 0.79, *R*^2^ = 0.63), and none of the predictors were significant (all *p*s > 0.20). Altogether, among all four handwriting tasks, only Horizontal Size in WS3 significantly predicted handwriting speed. These findings suggest that greater lateral movement during dictation may reduce handwriting efficiency in cognitively healthy older adults (Table 4).

**Table 4:** Multiple linear regression results for kinematic predictors of handwriting speed (WS1–WS4) in older adults without cognitive impairment.

Predictor	WS1 *b*	SE	*p*	WS2 *b*	SE	*p*	WS3 *b*	SE	*p*	WS4 *b*	SE	*p*
Intercept	131.15	61.58	0.040	9.73	16.49	0.566	−77.75	50.10	0.147	37.43	53.37	0.495
Vertical size	140.74	392.05	0.722	−239.47	192.24	0.237	237.29	273.90	0.403	269.49	319.09	0.414
Horizontal size	−285.18	233.42	0.230	125.21	168.06	0.471	**711.79**	194.88	**0.003**	83.09	150.76	0.591
Slant	31.64	34.18	0.361	−4.06	35.24	0.910	−22.09	23.94	0.374	−35.55	27.05	0.211
Start time	3.21	3.97	0.424	−3.89	2.26	0.111	−0.99	3.16	0.759	0.60	2.03	0.772
Duration	122.05	144.92	0.405	−46.25	81.05	0.579	76.81	121.46	0.539	−77.19	68.22	0.278
Relative pen-down duration	−55.86	73.08	0.450	92.35	55.96	0.125	17.60	54.25	0.751	37.72	58.39	0.530
Normalized y-Jerk	−0.03	0.02	0.228	0.02	0.03	0.440	−0.05	0.02	0.073	0.01	0.00	0.243
Average pen pressure	0.03	0.12	0.774	−0.02	0.07	0.801	0.04	0.08	0.632	−0.04	0.08	0.661
Number of strokes	−1.09	0.66	0.106	0.46	0.27	0.115	0.54	0.50	0.307	−0.04	0.34	0.904

WS1 = Copy; WS2 = Dictation; WS3 = Dictation; WS4 = Copy. Model fit: WS1 - *R* = 0.63, *R*^2^ = 0.40, *p* = n.s.; WS2 - *R* = 0.87, *R*^2^ = 0.76, *p* = n.s.; WS3 - *R* = 0.90, *R*^2^ = 0.81, *p* < 0.05; WS4 - *R* = 0.79, *R*^2^ = 0.63, *p* = n.s.

Bold values indicates statistically significant results (*p* < 0.05).

##### 3.2.2.2 Group with cognitive impairment

A series of multiple linear regression analyses was conducted to determine whether nine kinematic process variables—Vertical Size, Horizontal Size, Slant, Start Time, Duration, Relative Pen-Down Duration, Normalized y-Jerk, Average Pen Pressure, and Number of Strokes—could predict handwriting product performance in tasks WS1 through WS4.

For WS1 (copy), the model explained 40.1% of the variance (*R*^2^ = 0.40), but none of the individual predictors reached statistical significance (all *p*s > 0.25). In WS2 (dictation), the model accounted for 67.7% of the variance (*R*^2^ = 0.68), with two significant predictors: Start Time (*b* = −2.90, *p* = 0.046) and Number of Strokes (*b* = 0.63, *p* = 0.019). The WS3 (dictation) model explained 80.6% of the variance (*R*^2^ = 0.81). Three predictors were statistically significant: Vertical Size (*b* = 329.24, *p* = 0.033), Start Time (*b* = 5.51, *p* = 0.010), and Duration (*b* = −108.70, *p* = 0.029). Normalized y-Jerk approached significance (*p* = 0.051), suggesting a potential contribution to handwriting performance. For WS4 (copy), the model accounted for 82.7% of the variance (*R*^2^ = 0.83), but none of the predictors reached significance. However, Number of Strokes showed a trend toward significance (*b* = 0.90, *p* = 0.066). Taken together, these findings suggest that, unlike cognitively healthy individuals, older adults with cognitive impairment show more consistent relationships between kinematic process variables and handwriting outcomes—particularly in WS2 and WS3, where both temporal and spatial features significantly predicted handwriting performance (see Table 5).

**Table 5:** Multiple linear regression results for kinematic predictors of handwriting performance (WS1–WS4) in older adults with cognitive impairment.

Predictor	WS1 *b*	SE	*p*	WS2 *b*	SE	*p*	WS3 *b*	SE	*p*	WS4 *b*	SE	*p*
Intercept	66.37	179.18	0.718	3.93	25.95	0.882	26.21	23.17	0.282	17.76	28.80	0.555
Vertical size	−178.28	774.48	0.822	61.82	186.63	0.746	**329.24**	134.89	**0.033**	8.65	133.66	0.950
Horizontal size	250.56	504.62	0.628	83.99	80.99	0.320	88.01	77.41	0.280	121.92	101.11	0.262
Slant	−92.68	179.65	0.615	12.76	19.53	0.526	2.13	20.16	0.918	−13.18	16.35	0.444
Start time	4.21	10.96	0.707	**−2.90**	1.30	**0.046**	**5.51**	1.77	**0.010**	−4.06	2.72	0.175
Duration	312.48	379.10	0.426	10.55	53.49	0.847	**−108.70**	43.44	**0.029**	24.50	64.16	0.713
Relative pen-down duration	−222.90	196.58	0.279	30.53	37.46	0.431	16.32	23.34	0.499	−42.39	42.98	0.353
Normalized y-Jerk	−0.07	0.06	0.257	0.00	0.01	0.782	−0.01	0.01	0.051	0.00	0.00	0.638
Average pen pressure	0.16	0.25	0.525	−0.02	0.05	0.729	0.05	0.03	0.109	0.04	0.04	0.430
Number of strokes	−1.01	2.22	0.656	**0.63**	0.23	**0.019**	−0.47	0.25	0.088	0.90	0.42	0.066

WS1 = Copy; WS2 = Dictation; WS3 = Dictation; WS4 = Copy. Model fit: WS1 - *R* = 0.63, *R*^2^ = 0.40; WS2 - *R* = 0.82, *R*^2^ = 0.68; WS3 - *R* = 0.90, *R*^2^ = 0.81; WS4 - *R* = 0.91, *R*^2^ = 0.83.

Bold values indicates statistically significant results (*p* < 0.05).

## 4 Discussion

The present findings should be interpreted considering the theoretical framework outlined in the introduction, which conceptualizes handwriting as a hierarchical cognitive–motor activity in which task demands determine the degree of executive and working memory involvement. Specifically, pen-control tasks (DOTS and LINES) primarily index foundational graphomotor execution, whereas copying and, more markedly, dictation engages progressively greater linguistic and executive resources. Within this framework, we expected tasks with higher cognitive–motor demands to show greater sensitivity to cognitive impairment.

Consistent with this prediction, simple pen-control tasks did not meaningfully differentiate between cognitively impaired and cognitively healthy older adults. Although the omnibus model for LINES was statistically significant, no individual kinematic predictor independently contributed to diagnostic classification after correction for multiple comparisons. Moreover, process variables did not predict product outcomes in these tasks. These results suggest that isolated graphomotor execution—largely dependent on visuomotor coordination and basic motor planning—may not sufficiently tax working memory or executive systems to reveal early neurocognitive decline. This aligns with evidence indicating that cognitively mediated or dual-task paradigms are more sensitive to subtle deterioration than simple motor tasks (Yamada et al., 2022), despite the known utility of digital kinematic measures for detecting motor abnormalities (Impedovo et al., 2014).

In contrast, handwriting-speed tasks—particularly WS3 (dictation) and, to a lesser extent, WS4 (copy)—demonstrated stronger discriminatory capacity. Logistic regression analyses showed that temporal and stroke-organization variables, especially Duration and Number of Strokes, significantly increased the likelihood of cognitive impairment in WS3 and WS4. These findings support the notion that handwriting deficits become more pronounced when motor execution is tightly coupled with linguistic transformation and executive monitoring. Dictation tasks, which require phonological decoding, orthographic retrieval, serial maintenance, and motor programming, impose substantial demands on the phonological loop and central executive, as described in working memory models (De Vita et al., 2021; Olive and Kellogg, 2002). Under such conditions, reduced temporal efficiency and fragmented stroke production may reflect compromised motor planning and diminished executive control. This interpretation is consistent with prior work linking executive dysfunction and working memory deficits to reduced handwriting fluency in mild cognitive impairment and early dementia (Rosenblum et al., 2010; Rosenblum et al., 2013).

The differential pattern observed across groups further reinforces this interpretation. Among cognitively healthy older adults, the association between kinematic execution and handwriting speed was weak and largely non-significant. Even when regression models explained substantial variance (e.g., WS3), only Horizontal Size significantly predicted performance, suggesting that larger lateral amplitude was associated with slower writing. In the context of normal aging, such patterns may reflect adaptive or compensatory motor strategies that preserve functional output despite age-related neuromuscular slowing (Salthouse, 2019; Seidler et al., 2010). Similar dissociations between process and product measures have been reported in lifespan studies, in which functional performance remains relatively stable despite subtle kinematic alterations (Faundez-Zanuy et al., 2021).

An alternative explanation for differences in handwriting kinematics could involve non-cognitive factors common in older institutionalized populations, such as medication effects, general motor slowing, or frailty-related changes in neuromuscular function. Several precautions were taken to mitigate these potential confounders. Participants with neurological conditions known to directly affect motor control (e.g., stroke, Parkinson’s disease) were excluded, and both groups were recruited from similar institutional contexts, reducing environmental variability. Moreover, the absence of significant differences in the simple motor-control tasks (DOTS and LINES) suggests that basic graphomotor execution was largely preserved across groups. If medication or generalized motor impairment were the primary drivers of performance differences, similar effects would be expected across all tasks. Instead, group differences emerged mainly in tasks with higher cognitive–linguistic demands, supporting the interpretation that the observed patterns primarily reflect cognitive rather than purely motor influences.

By contrast, older adults with cognitive impairment showed a more consistent coupling between process and product variables, particularly in WS2 and WS3. In these tasks, Start Time, Vertical Size, Duration, and Number of Strokes emerged as significant predictors of handwriting performance. These findings indicate that motor initiation, spatial scaling, and temporal organization become increasingly constrained as cognitive resources decline. The near-significant contribution of Normalized y-Jerk in WS3 further suggests that reduced movement smoothness may compromise writing efficiency under higher cognitive load. This stronger process–product linkage in the cognitively impaired group supports the view that neurodegenerative processes reduce the availability of compensatory cognitive mechanisms, increasing reliance on core motor-execution parameters (Liu, 2011; Schiegg and Thorpe, 2017).

Importantly, these results converge with emerging research on digital biomarkers. Studies using machine learning approaches have demonstrated that spatiotemporal handwriting features are sensitive to pathological cognitive changes and can support classification of Alzheimer’s disease and related conditions (Diaz et al., 2021; Faundez-Zanuy et al., 2021; Demircioglu Diren, 2025; Nardone et al., 2025). The present findings extend this literature by showing that diagnostic sensitivity is task-dependent: only tasks that sufficiently engage executive and working memory systems reveal robust kinematic differences. This observation underscores the theoretical importance of selecting tasks with graded cognitive–motor demands, rather than relying exclusively on simple graphomotor paradigms.

From a clinical perspective, the findings highlight the potential of digitized dictation tasks as ecologically valid, low-cost tools for screening cognitive vulnerability in institutionalized older adults. Temporal efficiency and stroke organization appear to be particularly informative indicators. Furthermore, the stronger coupling between kinematic and product measures in cognitively impaired individuals suggests that handwriting analysis may not only aid early detection but also support monitoring of disease progression and intervention outcomes. Periodic assessment of kinematic parameters may facilitate individualized adjustment of therapeutic strategies and provide objective markers of change over time (Bisio et al., 2017).

Overall, the present study reinforces a hierarchical cognitive–motor model of handwriting in aging: basic motor tasks reflect foundational execution, whereas linguistically demanding tasks reveal the integrity of executive and working memory systems. Diagnostic differences emerged primarily under high cognitive–motor load, emphasizing that handwriting should be conceptualized not merely as a motor behavior, but as a dynamic behavioral marker of cognitive control processes in late life.

## 5 Limitations

Several limitations should be considered when interpreting the present findings. First, although major neurological and psychiatric conditions known to directly affect motor control were excluded, medication use was not systematically analyzed. Older institutionalized adults frequently receive pharmacological treatments (e.g., sedatives, antidepressants, antipsychotics) that may influence motor performance, potentially affecting handwriting kinematics. Future studies should incorporate detailed medication profiles and clinical indicators of frailty or motor functioning to further disentangle cognitive and motor contributions to handwriting performance. Second, the sample consisted exclusively of institutionalized older adults, which may limit the generalizability of the findings to community-dwelling populations with different health, functional, and environmental characteristics. Finally, the cross-sectional design restricts conclusions about the temporal progression of handwriting changes and their predictive value for cognitive decline. Longitudinal research is needed to determine whether the kinematic features identified in this study can serve as early markers of cognitive deterioration and to examine how handwriting performance evolves across different stages of cognitive impairment.

## 6 Practical implications

The present findings have important clinical and technological implications. First, handwriting-based assessments may serve as a non-invasive method for early detection of cognitive impairment in both institutional and primary care settings. Identifying specific deficits in timing and stroke organization provides actionable targets for motor-cognitive rehabilitation, particularly within occupational therapy and neurorehabilitation programs. Finally, the stronger association between kinematic features and handwriting performance observed in cognitively impaired individuals supports the use of tablet-based handwriting analysis in routine geriatric evaluations, potentially improving early diagnosis and ongoing monitoring of neurocognitive decline.

## 7 Conclusion

Grounded in a hierarchical cognitive–motor framework of handwriting, the present study demonstrates that kinematic features—particularly temporal efficiency and stroke organization—function as sensitive behavioral indicators of cognitive impairment in institutionalized older adults. Crucially, diagnostic differentiation emerged only under tasks imposing higher cognitive–motor demands, reinforcing the theoretical premise that handwriting becomes diagnostically informative when motor execution is tightly coupled with working memory, linguistic processing, and executive control.

Pen-control tasks that primarily assess basic graphomotor execution did not distinguish cognitive status, underscoring that isolated visuomotor coordination is insufficient to reveal subtle neurocognitive decline. In contrast, dictation and structured copying tasks, which require phonological transformation, orthographic retrieval, and sustained executive monitoring, revealed meaningful and consistent associations between handwriting process and product measures in individuals with cognitive impairment. This task-dependent sensitivity highlights the importance of selecting cognitively demanding paradigms when designing handwriting-based screening protocols.

The findings further indicate that, in cognitive impairment, the coupling between kinematic execution and functional writing outcomes becomes stronger and more systematic, suggesting reduced compensatory capacity and increased reliance on core motor–execution parameters. Within this context, digitized handwriting analysis emerges as a theoretically grounded and clinically relevant approach for detecting cognitive vulnerability.

Overall, digital handwriting assessment represents a low-cost, non-invasive, and ecologically valid method with strong potential for early screening and monitoring of cognitive decline. By integrating task complexity with fine-grained kinematic analysis, handwriting evaluation can move beyond simple motor assessment and serve as an accessible digital biomarker of executive and working memory integrity in aging populations.

## Data Availability

The raw data supporting the conclusions of this article will be made available by the authors, without undue reservation.
